# Hfq Globally Binds and Destabilizes sRNAs and mRNAs in Yersinia pestis

**DOI:** 10.1128/mSystems.00245-19

**Published:** 2019-07-16

**Authors:** Yanping Han, Dong Chen, Yanfeng Yan, Xiaofang Gao, Zizhong Liu, Yaqiang Xue, Yi Zhang, Ruifu Yang

**Affiliations:** a State Key Laboratory of Pathogen and Biosecurity, Beijing Institute of Microbiology and Epidemiology, Beijing, China; b Center for Genome Analysis, ABLife Inc., Wuhan, Hubei, China; c Laboratory for Genome Regulation and Human Health, ABLife Inc., Wuhan, Hubei, China; d Anhui Medical University, Hefei, Anhui, China; Princeton University

**Keywords:** CLIP-seq, Hfq, RNA degradation, *Y. pestis*, sRNA

## Abstract

Hfq is a ubiquitous Sm-like RNA-binding protein in bacteria involved in physiological fitness and pathogenesis, while its *in vivo* binding nature remains elusive. Here we reported genome-wide Hfq-bound RNAs in Yersinia pestis, a causative agent of plague, by using cross-linking immunoprecipitation coupled with deep sequencing (CLIP-seq) approach. We show that the Hfq binding density is enriched in more than 80% mRNAs of Y. pestis and that Hfq also globally binds noncoding small RNAs (sRNAs) encoded by the intergenic, antisense, and 3′ regions of mRNAs. An Hfq U-rich stretch is highly enriched in sRNAs, while motifs partially complementary to AGAAUAA and GGGGAUUA are enriched in both mRNAs and sRNAs. Hfq-binding motifs are enriched at both terminal sites and in the gene body of mRNAs. Surprisingly, a large fraction of the sRNA and mRNA regions bound by Hfq and those downstream are destabilized, likely via a 5′P-activated RNase E degradation pathway, which is consistent with a model in which Hfq facilitates sRNA-mRNA base pairing and the coupled degradation in Y. pestis. These results together have presented a high-quality Hfq-RNA interaction map in Y. pestis, which should be important for further deciphering the regulatory role of Hfq-sRNAs in Y. pestis.

**IMPORTANCE** Discovered in 1968 as an Escherichia coli host factor that was essential for replication of the bacteriophage Qβ, the Hfq protein is a ubiquitous and highly abundant RNA-binding protein in many bacteria. With the assistance of Hfq, small RNAs in bacteria play important roles in regulating the stability and translation of mRNAs by base pairing. In this study, we want to elucidate the Hfq-assisted sRNA-mRNA regulation in Yersinia pestis. A global map of Hfq interaction sites in Y. pestis was obtained by sequencing cDNAs converted from the Hfq-bound RNA fragments using UV cross-linking coupled immunoprecipitation technology. We demonstrate that Hfq could bind to hundreds of sRNAs and the majority of mRNAs in Y. pestis. The enriched binding motifs in sRNAs and mRNAs are complementary to each other, suggesting a general base-pairing mechanism for sRNA-mRNA interaction. The Hfq-bound sRNA and mRNA regions were both destabilized. The results suggest that Hfq binding facilitates sRNA-mRNA base pairing and coordinates their degradation, which might enable Hfq to surveil the homeostasis of most mRNAs in bacteria.

**Author Video**: An author video summary of this article is available.

## Abstract (precis)

Discovered in 1968 as an Escherichia coli host factor that was essential for replication of the bacteriophage Qβ, the Hfq protein is a ubiquitous and highly abundant RNA-binding protein in many bacteria. With the assistance of Hfq, small RNAs in bacteria play important roles in regulating the stability and translation of mRNAs by base pairing. In this study, we want to elucidate the Hfq-assisted sRNA-mRNA regulation in Yersinia pestis. A global map of Hfq interaction sites in Y. pestis was obtained by sequencing cDNAs converted from the Hfq-bound RNA fragments using UV cross-linking coupled immunoprecipitation technology. We demonstrate that Hfq could bind to hundreds of sRNAs and the majority of mRNAs in Y. pestis. The enriched binding motifs in sRNAs and mRNAs are complementary to each other, suggesting a general base-pairing mechanism for sRNA-mRNA interaction. The Hfq-bound sRNA and mRNA regions were both destabilized. The results suggest that Hfq binding facilitates sRNA-mRNA base pairing and coordinates their degradation, which might enable Hfq to surveil the homeostasis of most mRNAs in bacteria.

## INTRODUCTION

Hfq is a small, ubiquitous, and highly abundant RNA-binding protein in bacteria involved in physiological fitness and pathogenesis (1). It forms a doughnut-like homohexameric structure and belongs to the Sm/LSm superfamily (2, 3). Similar to its eukaryotic counterparts, each monomer carries the signature Sm motif for protein-protein interaction and RNA binding that contributes to posttranscriptional regulation (2, 4, 5). Hfq plays a central role in mRNA stability regulation by cooperating with regulatory RNAs in bacteria (6–8). The largest class of regulatory RNAs in bacteria is small RNAs (sRNAs), including *cis*-encoded antisense sRNAs and *trans*-encoded sRNAs. Both subclasses of sRNAs are capable of causing translation inhibition, mRNA cleavage, or degradation (9). Hfq binds to sRNAs and promotes the limited base pairing with their mRNA targets (2–7, 9–11). Hfq-promoted sRNA binding target sites are not only located in the canonical Shine-Dalgarno SD/AUG region, but also in other regions of the target mRNAs (12–14).

Hfq is known to bind most sRNAs via its proximal face binding the poly(U) sequences typical of rho-independent terminators, with uridine stacked in pockets between neighboring monomers around the central pole (11, 15–18). The distal face prefers A-rich sequences in Hfq-interacting mRNAs and sRNAs (19–21). It has been proved that the rim (lateral face) of Hfq contacts UA-rich sequences in sRNAs and mRNAs (11, 22–26). The multiple binding surfaces of an Hfq homohexamer enable a large flexibility of this protein in mediating not only the sRNA-mRNA interaction but probably also the sRNA-sRNA interactions in regulating the stabilities of both types of bacterial RNAs (11, 27). Genome-wide analysis of Hfq-bound RNA fragments in Escherichia coli and Salmonella enterica serotype Typhimurium has provided the global binding motifs of Hfq on sRNAs and mRNAs (27–29).

A key endoribonuclease for RNA processing and decay in *Gammaproteobacteria* is RNase E, which recognizes its substrates via two different modes of action (30). RNase E senses and binds the 5′-monophosphate group of a target, which enables the enzyme to distinguish and prefer cleaved RNAs that have already undergone at least one RNase cleavage from the primary transcript with a 5′-triphosphate group. The cleavage sites activated by the 5′-monophosphate binding can be downstream (31). Alternatively, RNase E directly makes a cut in the body of an mRNA (4, 5, 30). RNase E is crucial for sRNA-induced decay of target mRNAs and sRNAs themselves (4, 30–32). RNase E-induced degradation of sRNAs can occur either when they are free or when they are paired with their targets. The latter is called coupled degradation (32–34). RNase E contains a defined region for Hfq binding (35). The interaction between RNase E and Hfq seems to play an important role in sRNA-mediated mRNA decay (4, 30, 36). However, it is unclear how much this potential novel type of “degradosome” acts in controlling bacterial mRNA decay.

Plague caused by Yersinia pestis is a zoonotic disease primarily transmitted between fleas and mammals. Hfq has been found to be required for the virulence of Y. pestis and other *Yersinia* species (37, 38). The Y. pestis Hfq protein has ∼85% similarity with their homologues in E. coli and *S.* Typhimurium and 100% sequence identity within the Sm1 and Sm2 signature regions (2). However, its role in RNA regulation is not well studied in Y. pestis.

In this study, we sequenced and analyzed cDNA libraries generated from the Hfq-bound Y. pestis RNA fragments using two different UV cross-linking and immunoprecipitation methods, one resembling cross-linking immunoprecipitation coupled with deep sequencing (CLIP-seq) (29) and another resembling RNA immunoprecipitation coupled to sequencing (RIP-seq) (39). Considering that Hfq normally binds to a single-stranded region near a hairpin structure, we mildly digested Hfq-bound RNA molecules in this study to recover RNA segments containing Hfq-bound sites. Using transcriptome sequencing data as controls, we showed that Hfq binds most of the expressed mRNAs and sRNAs in Y. pestis. Hfq binds mRNAs not only via known motifs but also via the novel G-rich motifs. Moreover, we demonstrated an increased destabilization of RNA segments that are bound by Hfq, irrespective of whether they are located in sRNAs or mRNAs. The results suggest that the Hfq-facilitated sRNA-mRNA base pairing might be more likely coupled with their degradation than previously appreciated.

## RESULTS

### Global binding profiles of Hfq in Y. pestis by CLIP-seq.

To globally map Hfq-binding RNAs and Hfq-binding sites in Y. pestis cells, we used two strains expressing a FLAG epitope. The experimental Hfq strain expressed Hfq-FLAG from a plasmid in a *hfq* deletion (Δ*hfq*) genetic background (Hfq-FLAG). A wild-type (WT) strain with Flag epitope (WT-FLAG) was used as a control. Another control by transforming pHfq into a Δ*hfq* strain (Hfq) was also obtained. A previous study has shown that the exogenously expressed Hfq-FLAG was functionally competent (38). Using native polyacrylamide gel electrophoresis (PAGE), we found that Hfq-FLAG stably existed as trimer and hexamer in bacterial cells (see Fig. S1A in the supplemental material). Transcriptome profiling of the constructed strains demonstrated a highly correlated expression pattern (*R *>* *0.98) of these three strains (Fig. S1B).

10.1128/mSystems.00245-19.1 FIG S1 Experimental procedure and Hfq-bound gene profile of CLIP-seq. (A) PAGE gel electrophoresis of cellular Hfq on Hfq-FLAG strain under nondenaturing conditions followed by Western blot analysis. 25 mM Tris-HCl (pH 8.0) was used as a control. (B) Gene expression correlation analysis between Hfq-FLAG and WT-FLAG by Pearson correlation. (C) Experimental procedure of CLIP-seq and RNA-seq. (D) Western blot of Hfq-RNA complex separated by SDS-PAGE by using anti-FLAG monoclonal antibody (left) and electrophoresis visualization of PCR products amplified from cDNA before (middle) and after (right) gel-cutting purification. (E) Enriched functional KEGG pathways of Hfq-bound and -unbound genes under *in vitro* vegetative growth conditions. Download FIG S1, TIF file, 4.7 MB. Copyright © 2019 Han et al. 2019 Han et al. This content is distributed under the terms of the Creative Commons Attribution 4.0 International license.

**FIG 1:**
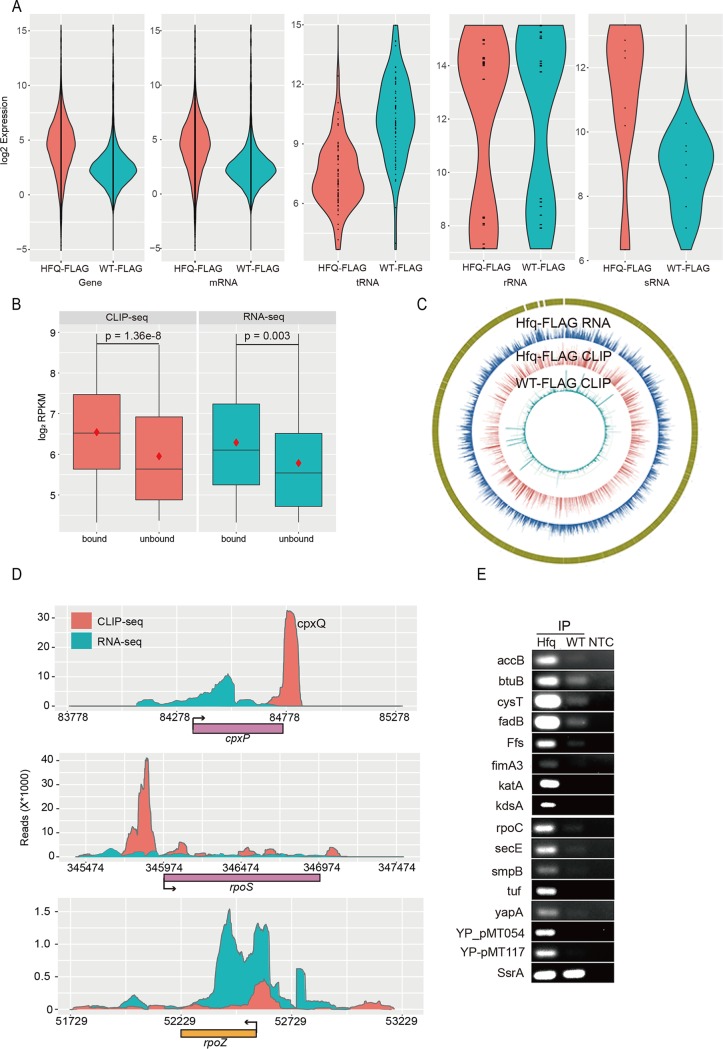
CLIP-seq revealed that Hfq binds more than 80% of transcribed RNAs in Y. pestis. (A) Violin plot of Hfq binding profiles for all genes, mRNA, tRNA, rRNA, and seven annotated sRNAs. Red violins represent the Hfq-FLAG strain, and blue violins represent the WT-FLAG strain. (B) Box plot of gene expression level classified by Hfq binding. The CLIP-seq data (orange) and the RNA-seq data (blue) are indicated. The red diamonds represent mean values. The *y* axis represents the log_2_ detection level (RPKM) of genes. (C) Read density presentation of the whole genome for three sequencing samples, RNA-seq of Hfq-FLAG, CLIP-seq of Hfq-FLAG, and CLIP-seq of WT-FLAG, from external to internal, respectively. (D) Read density plot for Hfq-bound *cpxP* and *rpoS* mRNA and for Hfq-unbound *rpoZ* in Y. pestis. Hfq binding density (orange) and RNA expression density (blue) are shown. (E) RIP-PCR validation of Hfq-bound sRNA and mRNAs. NTC, no template control.

The FLAG-tagged Hfq-RNA complexes were cross-linked by UV irradiation of cultured cells, followed by coimmunoprecipitation by anti-FLAG and partial digestion of unprotected RNA segments by RNase T1 (Fig. S1C). The Hfq-bound RNA segments were purified and ligated with adaptors for sequencing (Fig. S1D). Equal amounts of bacterial cultures from ΔHfq_Hfq-FLAG and WT-FLAG strains were lysed and subjected to parallel immunoprecipitation experiments to obtain CLIP-seq data. Experiments with these two strains were strictly performed in parallel. However, we obtained much less cDNAs from the WT-FLAG strain than from the Hfq-FLAG strain, suggesting that the FLAG tag itself did not yield much RNA binding noise and therefore was successful coimmunoprecipitation (co-IP) system (Fig. S1D). We obtained 18.1 million Hfq-FLAG-bound RNA tags and 2.1 million FLAG-bound control tags and then mapped them to the Y. pestis 91001 genome (see Table S1 in the supplemental material). Free FLAG control exclusively bound rRNA (86.03%) and tRNA (2.38%), indicating nonspecific binding. The fraction of CLIP-seq reads mapped to the annotated sRNA regions from the Hfq-FLAG strain was 10-fold higher than that of the WT-FLAG control, consistent with the specific sRNA binding activity of Hfq. We found the increased Hfq binding in sRNA, mRNA, and intergenic regions seems to be genome-wide rather than to some specific genes (Fig. 1A). These results suggest that Hfq selectively binds a large population of sRNA and mRNA in bacterial cells.

10.1128/mSystems.00245-19.5 TABLE S1 Mapping of clean reads on the reference genome. Download Table S1, DOCX file, 0.01 MB. Copyright © 2019 Han et al. 2019 Han et al. This content is distributed under the terms of the Creative Commons Attribution 4.0 International license.

To identify the Hfq-bound genes from our CLIP-seq data, we normalized the CLIP-seq reads in each gene to the nonspecific bound 23S rRNA gene *YP_r2*. This rRNA represents the most abundant one among all identified RNAs in both strains. With twofold enrichment and at least 10 bound reads as thresholds, we obtained a total of 3,331 Hfq-bound RNAs and 864 Hfq-unbound RNAs in Y. pestis (Table S2A and B). All of the 22 rRNA genes and 65 out of 68 tRNA genes were not bound to Hfq. Among the seven annotated sRNAs, six were detected with the CLIP-seq reads, four were identified as Hfq bound, and two were not bound to Hfq (Table S2C). The Hfq-bound sRNAs includes the well-studied Spf, CsrB, and SsrS. In previous studies (40, 41), Spf sRNA is Hfq bound in both E. coli and *Salmonella*. Ffs, SsrA, RnpB, and SsrS are not bound by Hfq in E. coli, and CsrB and SsrS are not Hfq bound in *Salmonella*. The Hfq-unbound sRNAs in Y. pestis included Ffs, SsrA, and RnpB (Table S2C).

10.1128/mSystems.00245-19.6 TABLE S2 Tables of Hfq-bound and -unbound genes and the related binding ratio. Download Table S2, XLSX file, 0.3 MB. Copyright © 2019 Han et al. 2019 Han et al. This content is distributed under the terms of the Creative Commons Attribution 4.0 International license.

We showed that 80.5% (3,323 out of 4,128) of all mRNA genes were enriched in the Hfq-FLAG strain. Transcriptome sequencing data from the two experimental Y. pestis strains cultured under the same condition were obtained as another set of controls (Table S1). The CLIP-seq method revealed that Hfq-bound and -unbound genes were well expressed, and it seemed that Hfq-bound genes tend to be clustered in the higher-expressed gene population (Fig. 1B). Hfq-bound genes were enriched in a large array of metabolic pathways, while Hfq-unbound genes were enriched in flagellar assembly, bacterial secretion system, and chemotaxis (Fig. S1D). These results collectively suggested that Hfq binds to most genes important for the exponential growth of Y. pestis, which supports its global and extensive regulatory role.

Comparison between Hfq binding profiles and the corresponding transcriptional profiles by counteracting common depth from each other indicated the binding specificity (Fig. 1C). For example, the CLIP-seq and transcriptome sequencing (RNA-seq) reads peaked at different locations for the previously known Hfq-bound *cpxP* mRNA. The RNA-seq reads were spread throughout the coding region, while CLIP-seq reads peaked at the 3′ untranslated region (3′UTR) corresponding to the CpxQ sRNA (Fig. 1D, top). The Hfq-bound CpxQ sRNA has been recently reported to play a role in protecting bacteria against inner membrane damage (42). The 5′ leader of *rpoS* mRNA is located at the 3′ part of *nlpD* mRNA and is known to be bound by Hfq and DsrA sRNA (17, 43). We showed that Hfq has a strong binding peak in the 5′ leader region of *rpoS* mRNA. Moderate binding peaks in the gene body region and 3′ downstream region were also evident (Fig. 1D, middle). The CLIP-seq and RNA-seq profiles of one Hfq-unbound mRNA, *rpoZ*, were also shown (Fig. 1D, bottom).

We validated the Hfq-bound mRNAs and sRNAs obtained above using RNA immunoprecipitation and PCR (RIP-PCR) experiment (See methods). After coimmunoprecipitation by anti-FLAG using Hfq-FLAG and WT-FLAG cell lysates, we used SsrA sRNA as an unbound control to validate 15 randomly chosen Hfq-bound sRNAs and mRNAs, including Ffs, BtuB, CysT, FadB, FimA3, KatA, KdsA, SecE, SmpB, Tuf, YapA, YP-PMT054, and YP-PMT117. All of these Hfq-bound and unbound sRNAs and mRNAs were validated (Fig. 1E).

### Potential regulatory RNAs predicted from CLIP-seq and RNA-seq.

**FIG 2:**
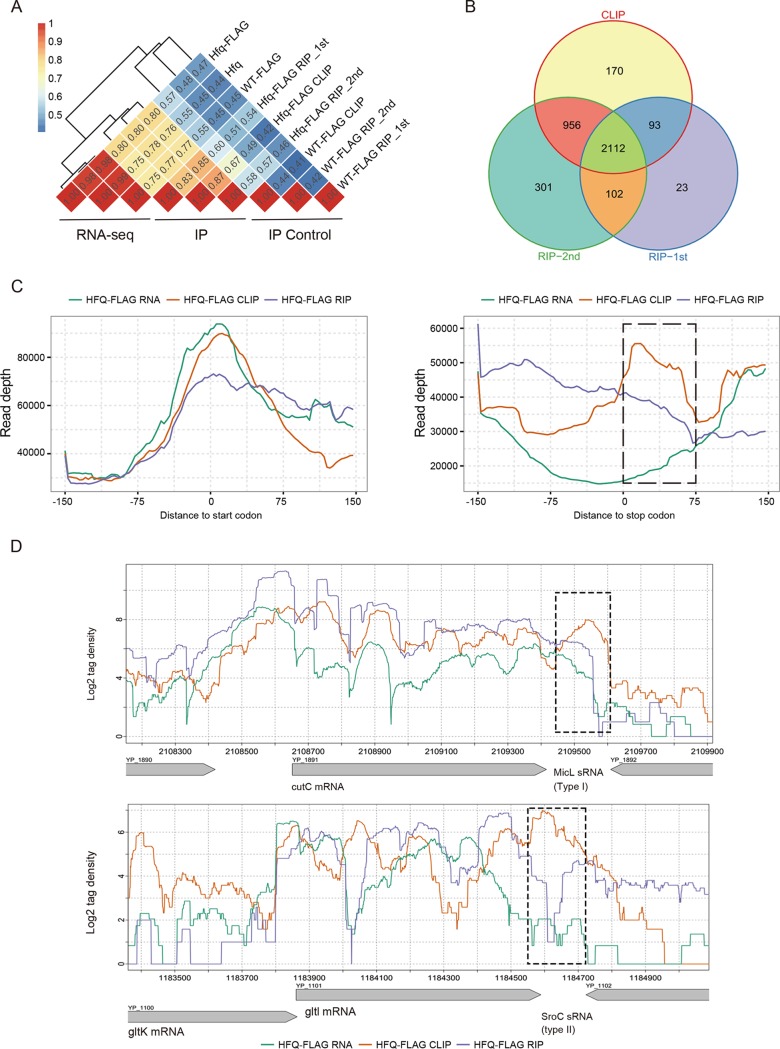
RIP-seq experimental confirmation of the global binding properties of Hfq. (A) Pearson correlation analysis of detected gene abundance from RNA-seq (one set), CLIP-seq (one set), and RIP-seq (two sets) experiments using different strains. RNA-seq was performed with three strains. CLIP-seq and RIP-seq were performed with two strains (Hfq-FLAG and WT-FLAG), and WT-FLAG was used as a control. (B) Venn diagram showing the overlapping Hfq-bound mRNAs and sRNAs among three different immunoprecipitation experiments. (C) Read distribution around the 5′ leader region and the 3′ region from the CLIP-seq and RNA-seq data and one set of RIP-seq data. The left panel shows 5′ leader regions, and the right panel shows 3′ leader regions. The black dashed box indicates the region downstream of the stop codon and enriched in CLIP peak. (D) Read density illustration of two major sRNA types transcribed from the 3′ region of mRNA. The top panel shows a type I sRNA, and the bottom panel shows a type II sRNA. The black dashed box shows the locations of predicted sRNAs.

To more globally validate the CLIP-seq results, we performed two independent sets of Hfq RIP-seq experiments with the same strains and growth conditions. A total of 9,186,327 and 26,971,805 clean reads were obtained from the Hfq-FLAG strains. The mapping features of RIP-seq data were similar to those of the CLIP-seq data (Table S1). Moreover, distribution of RIP-seq reads in all genes was more similar among the two repeated experiments and the CLIP-seq data compared to their RNA-seq and WT-FLAG controls (Fig. 2A and Fig. S2A). When Hfq-bound and -unbound genes were similarly identified from the two sets of RIP-seq data, the results showed that 3,263 (86.85%) Hfq-bound mRNAs and sRNAs overlapped among different immunoprecipitation experiments (Fig. 2B).

10.1128/mSystems.00245-19.2 FIG S2 Sample correlation and sRNA prediction of CLIP-seq and RNA-seq data. (A) Three-dimensional correlation of the read density mapped onto all Y. pestis genes from CLIP-seq, RIP-seq, and RNA-seq experiments. (B) Hfq binding of the intergenic and antisense regions. Bar plot presentation of the results from analysis of CLIP-seq and RIP-seq 2nd data. (C) Read density illustration of Y. pestis known sRNAs in CLIP-seq, RIP-seq, and RNA-seq experiments, including Spf, SsrS, CsrB, Ffs, MicF, and RnpB. A plus or minus sign behind experiment represents the detection state of sRNA in each experiment. Download FIG S2, TIF file, 4 MB. Copyright © 2019 Han et al. 2019 Han et al. This content is distributed under the terms of the Creative Commons Attribution 4.0 International license.

In order to better understand the length features of Hfq-bound intergenic and antisense RNAs revealed by CLIP-seq data, longer Hfq-bound RNA segments (with mean insertion size of 150 nucleotides [nt]) were selected for sequencing in RIP-seq experiments shown in this study. Without RNase T1 digestion, RIP-seq methodology preferred longer transcripts and selected against short sRNA transcripts. We found that 67.5% intergenic regions and 70.3% antisense regions showed Hfq-bound evidence from CLIP-seq data, while 49.1% and 30.4% corresponding regions obtained Hfq-bound evidence from one set of RIP-seq data (Fig. S2B). We also found that two out of three Hfq-bound sRNAs from CLIP-seq lost Hfq-bound signals from RIP-seq (Fig. S2C). Compared with the CLIP-seq binding profile, the specifically reduced binding capacity in the intergenic and antisense RNAs, but not mRNAs from RIP-seq libraries, suggests that intergenic and antisense RNAs are generally short transcripts. Their higher Hfq-bound efficiency indicates an unexpected global function in gene regulation.

The 5′ leaders are well-known to regulate bacterial gene expression (9). The regulatory role of the 3′ region of mRNA genes has recently been identified (44). We explored the Hfq binding profiles in these two classes of noncoding regions in Y. pestis. Compared to the 5′ leader regions (Fig. 2C, left), we showed that Hfq binding is strongly enriched at the 3′ regions of downstream mRNA genes, which was evident both by CLIP-seq and RIP-seq density (Fig. 2C, right), suggesting a global regulatory role of 3′ region in Y. pestis.

The mRNA 3′ regions have been reported to encode two major types of sRNAs. Type I is independently transcribed from the 3′ end of a mRNA, and type II is processed by an endonuclease at the 3′ region of the mRNA from a primary transcript (44, 45). The four reported type I and II sRNAs from E. coli and *Salmonella* were examined for their transcripts and Hfq binding profiles in Y. pestis. Three sRNAs’ host mRNAs were well expressed in Y. pestis, including the type I MicL in *cutC* mRNA and type II SroC and CpxQ sRNAs located at the 3′ of *gltl* and *cpxP* mRNA. All of these three 3′ sRNAs were bound by Hfq (Fig. 2D and Fig. 1D).

### Hfq binding sites and motifs in the coding and noncoding regions.

**FIG 3:**
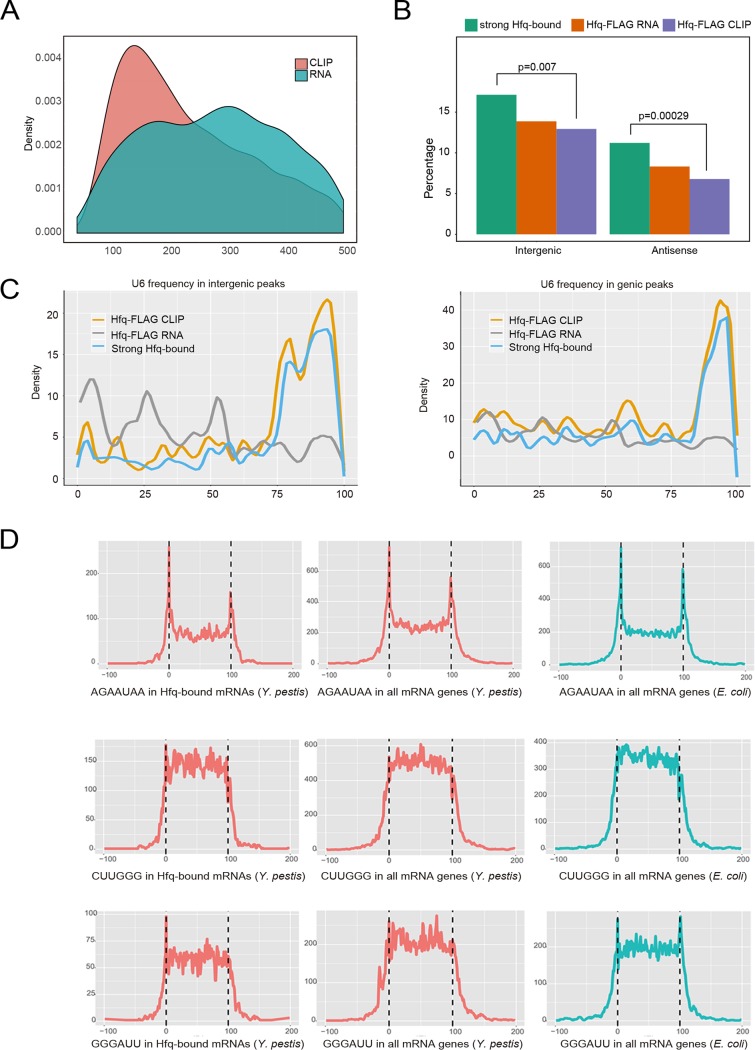
Hfq binding site and motif distribution in the coding and noncoding regions. (A) Peak length distribution of CLIP-seq and RNA-seq experiments. (B) Bar plot showing the percentage of intergenic and antisense peaks in RNA-seq, CLIP-seq, and strong Hfq-bound peaks. The *P* values (Fisher’s exact test) are shown to illustrate the enrichment of peaks in the antisense and intergenic regions. (C) U_6_ stretch frequency and position in three classes of peaks, including all CLIP-seq peaks, all RNA-seq peaks, and strong Hfq-bound peaks. Presentation was separated by intergenic (left) and genic (right) regions. (D) Motif distribution along the Hfq-bound mRNAs (left), all mRNAs of Y. pestis (middle), and in all mRNAs of E. coli (right).

We used a window-based algorithm, calculating read density in adjacent windows and comparing their difference between IP and control to detect the Hfq-binding and transcriptional peaks from CLIP-seq and RNA-seq, respectively (see Materials and Methods for detailed information). A total of 2,511 and 1,518 peaks were recovered from CLIP-seq and RNA-seq data, respectively (Table S3). The dominant length of Hfq-bound peaks was around 150 nt and could be as long as 500 nt (Fig. 3A). Such a long Hfq-binding region could be partially resulted from the partial RNase T1 digestion during operation. In contrast, the transcript peaks were generally longer than Hfq-bound peaks (Fig. 3A).

10.1128/mSystems.00245-19.7 TABLE S3 Peak summary and classification by region. Download Table S3, DOCX file, 0.02 MB. Copyright © 2019 Han et al. 2019 Han et al. This content is distributed under the terms of the Creative Commons Attribution 4.0 International license.

Theoretically, CLIP-seq peaks indicated the Hfq-bound regions, while RNA-seq peaks indicated the steady-level transcripts. The latter is expected to cover the former. We then selected Hfq-bound peaks in which CLIP peaks containing fourfold-more CLIP-seq reads than RNA-seq reads, resulting in 1,499 qualified peaks. These peaks were defined as strong peaks, and other peaks from CLIP-seq were defined as weak peaks. Among these peaks, 1,168 overlapped the known genes, 131 overlapped the antisense strands, and 200 overlapped in the intergenic regions (Fig. 3B), showing that Hfq has a larger tendency to associate with the noncoding regions, including both the intergenic and antisense regions. The selection criteria are quite strict, as reflected by the loss of four of the five Hfq-bound sRNAs and all three Hfq-bound tRNAs identified above (Fig. 1). This strict selection should allow us to explore the reliable binding features, particularly binding motifs of Hfq in Y. pestis transcriptome.

We used Homer software (46), well suited for finding motifs in large-scale genomics data, to recover highly represented Hfq binding motifs from these three different classes of peaks. These cellular motifs harbor all three known types of motif sequences, including poly(U), A-rich, and UA-rich bound on the proximal, distal, and rim surfaces of Hfq, respectively. Hfq-bound RNA motifs in Y. pestis were conserved in short motif sequence composition but quite flexible in motif organizations. For example, the top motif AAUAA was highly represented in mRNAs, intergenic RNAs, and antisense RNAs (Table 1). The two conserved nucleotides preceding this motif were AG(C) in mRNAs, AG in intergenic sRNAs, and UA in antisense sRNAs. The resulting motif composition contained a combination of ARN and UAA motifs in mRNAs and intergenic sRNAs, and two UAA motifs in antisense sRNAs. Moreover, our results revealed a previously unrecognized G-rich motif. The GGGGAUU motif was highly represented in Hfq-bound mRNAs and intergenic sRNAs, but not in antisense sRNAs. The G-rich motif might contact Hfq at the distal face as the ARN motif does (20).

**TABLE 1:**
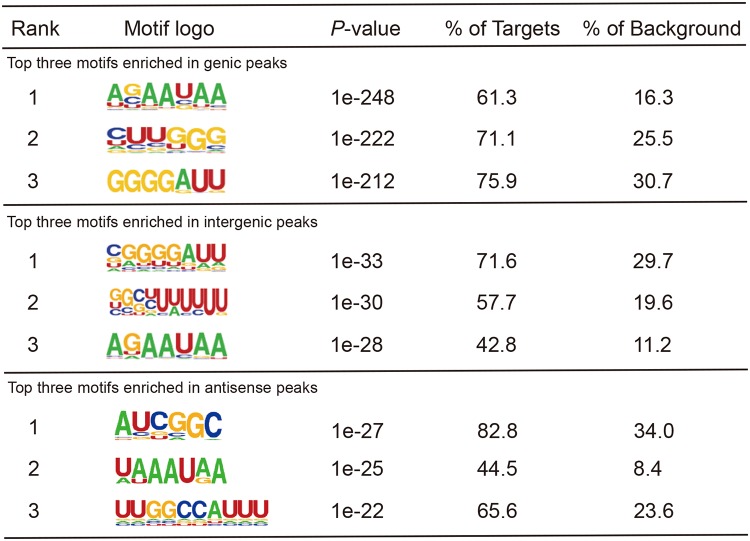
Top three consensus motifs generated from three kinds of peaks bound by Hfq^*a*^

a The percentages of target or background represent the detection ratio (as a percentage) of the motifs in Hfq-bound peaks or simulated background peaks from randomly selected genomic sequences, respectively.

As a conserved sequence component of the rho-independent terminator, poly(U) is a symbol of the Hfq-bound sRNAs. We found that the U_6_ stretch motif was presented in 57.7% of the intergenic peaks (Table 1). As expected, the U_6_ stretch was preferentially located at the 3′ ends of strong Hfq-bound peaks (Fig. 3C, left). No such enrichment was observed for RNA-seq peaks (Fig. 3C, left). A population of Hfq-bound mRNA peaks also contain the U_6_ motif at the 3′ end (Fig. 3C, right). Such a U_6_ stretch enrichment at the 3′ end was not much evident for the antisense RNAs (Fig. S3A). It is noteworthy that the U_5_ motif occurred at a much higher frequency with a pattern similar to the U_6_ stretch (Fig. S3B to D). The presence of the poly(U) motif at the 3′ end of Hfq-bound mRNA suggests that Hfq could use its proximal surface to contact with mRNA as well, consistent with the recently identified class of sRNAs located in the 3′ regions of mRNAs.

10.1128/mSystems.00245-19.3 FIG S3 Motif distribution and sRNA annotation on predicted peaks in CLIP-seq and RNA-seq. (A) U_6_ stretch frequency and position in antisense peaks of three samples, including CLIP-seq, RIP-seq, and RNA-seq (top). (B) U_5_ stretch frequency and position in intergenic peaks of the three samples. (C) U_5_ stretch frequency and position in antisense peaks of the three samples. (D) U_5_ stretch frequency and position in genic peaks of the three samples. (E) Venn diagram of the overlap among three different studies (47–49). (F) Promoter and terminator prediction of sRNAs from CLIP-seq and RNA-seq data. sRNAs were classified into intergenic and antisense sRNAs. Download FIG S3, TIF file, 2.2 MB. Copyright © 2019 Han et al. 2019 Han et al. This content is distributed under the terms of the Creative Commons Attribution 4.0 International license.

In addition, we analyzed the distribution of the top three motifs on mRNAs harboring strong Hfq-bound sites. The AG/CAAUAA motif was found most often at the 5′ and 3′ ends of the target mRNAs, while the other two highly represented motifs CUUGGG and GGGAUU were presented in the body regions of mRNAs (Fig. 3D, left panels). We wondered whether the motif selection was caused by Hfq selection. Analysis of the sequence composition of all mRNAs from both Y. pestis and E. coli showed that the above motif patterns were true for all mRNAs (Fig. 3D, middle and right panels). Therefore, the location specificity of these Hfq-bound mRNA motifs should not be caused by selection of Hfq binding; instead, it is an intrinsic feature of bacterial mRNA structure. Nevertheless, we noticed that all classes of motifs located at the 5′ end were more selected than those located at other regions (Fig. 3D, left and middle panels). We found that 53.64% and 70.83% mRNAs from Y. pestis and E. coli, respectively, contain either an AGAAUAA, CUUGGG, or GGGAUU motif. Of Hfq-bound mRNAs, 71.82% contain at least one of these top motifs. AGAAUAA motifs at the 5′ and 3′ termini were present at similarly high frequencies in both Y. pestis and E. coli.

### The distinct sRNA profile between CLIP-seq and RNA-seq.

We wanted to identify Y. pestis sRNAs to further understand the binding features of Hfq-sRNA by using the RNA-seq data obtained from the same bacterial strains and similar culture conditions as for generating CLIP-seq data. Although previous studies have identified hundreds of *Yersinia* sRNAs, these identified sRNAs do not overlap completely among different studies, even for the same species (47–50) (Fig. S3E).

**FIG 4:**
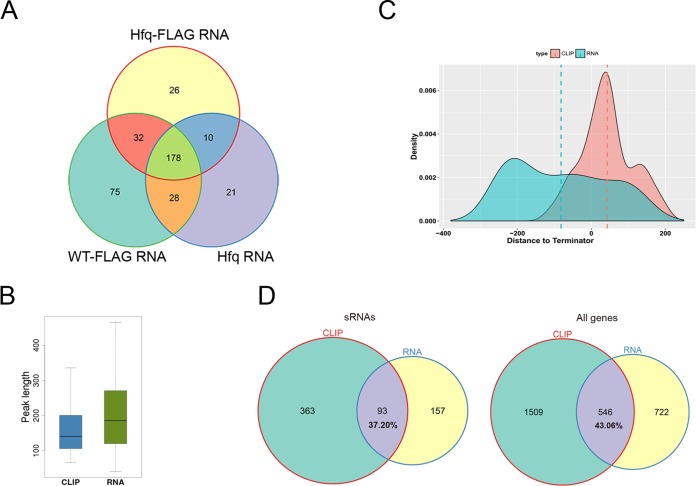
Candidate sRNAs identified by RNA-seq and CLIP-seq. (A) Venn diagram showing the overlap of sRNA predicted from RNA-seq data of three different Y. pestis strains. (B) Box plot of the length of sRNA peaks obtained from CLIP-seq and RNA-seq data. (C) Density plot of the distance distribution of peak end to terminator. The dashed lines represent the average center of predicted sRNAs. (D) Venn diagram showing the overlap between CLIP-seq and RNA-seq peaks. We showed sRNA peaks (left panel) and overall peaks (right panel).

We predicted sRNAs from RNA-seq data in intergenic and antisense regions by using the peak calling algorithm described above. We identified 250, 315, and 238 transcriptional peaks in the Hfq-FLAG strain, WT_FLAG strain, and Hfq strain, respectively (Table S3). These peaks strongly overlapped each other, and 178 of them have more than 80% overlapped sequence, which were considered the same sRNAs (Fig. 4A). After merging, 373 sRNA transcripts were identified from all three strains. Among the intergenic sRNAs, only 40 of them harbor a canonical terminator within 150 nt of their 3′ ends, while 85 harbored a canonical promoter within 150 nt of their 5′ end (Fig. S3F). Among them, 12 harbor both the terminator and promoter. More than 40% of the previously identified *Yersinia* sRNAs from different studies were found among these 373 sRNAs (Table S4).

10.1128/mSystems.00245-19.8 TABLE S4 Homology analysis of sRNAs from different studies. Download Table S4, DOCX file, 0.01 MB. Copyright © 2019 Han et al. 2019 Han et al. This content is distributed under the terms of the Creative Commons Attribution 4.0 International license.

We also predicted 456 qualified intergenic and antisense peaks bound by Hfq from CLIP-seq data (Table S3), with a shorter length distribution than that from RNA-seq (Fig. 4B, *P* value = 2.42e−9 by *t* test). Compared to the RNA-seq sRNA peaks, Hfq-bound sRNA peaks were closer to canonical transcription terminators, and most of them were located downstream of the predicted terminators (Fig. 4C). When we analyzed whether Hfq-bound sRNA peaks and RNA-seq sRNA peaks overlapped by setting 1-nucleotide overlap as a criterion, i.e., genomic overlap of ≥1 nt, about two-thirds of Hfq-bound sRNA peaks did not overlap with RNA-seq sRNA peaks (Fig. 4D). These results implied the inconsistent features of peaks predicted by CLIP-seq and RNA-seq data, which led to a hypothesis that Hfq binding may induce destabilization of sRNAs and mRNAs, rendering Hfq-bound sRNAs regions less detectable than the unbound regions by the RNA-seq approach.

### RNA segments downstream of Hfq-bound sites in both sRNAs and mRNAs were destabilized.

**FIG 5:**
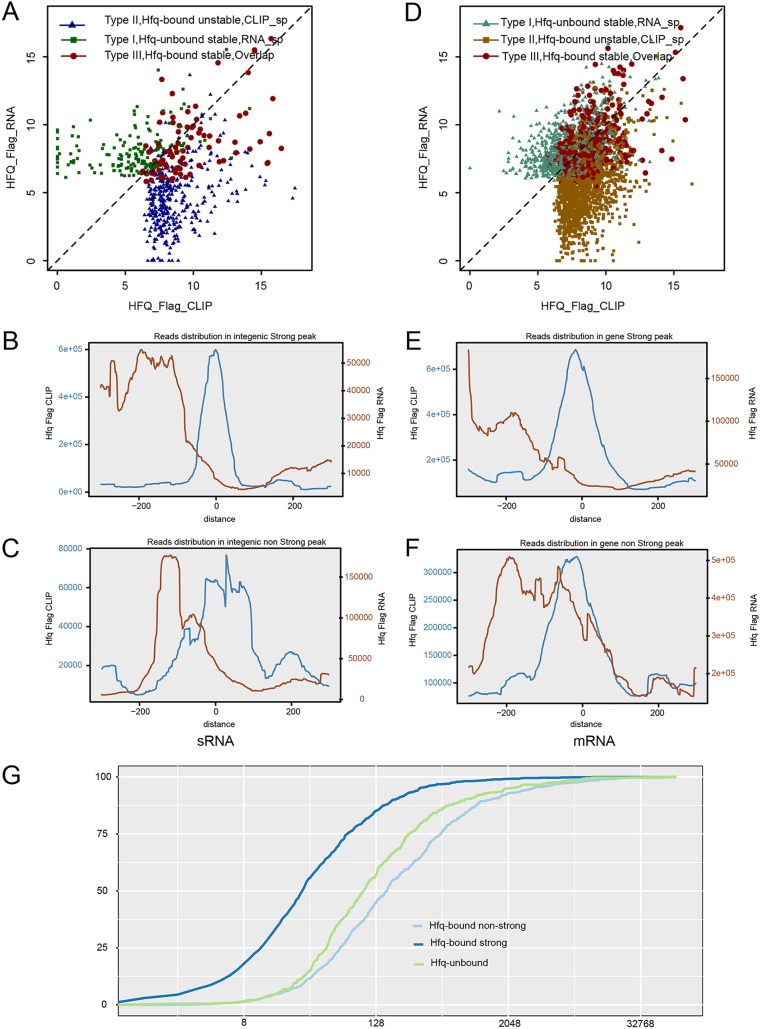
Hfq-bound sRNA and mRNA segments were generally unstable. (A) Dot plot of the abundance of three classes of sRNA peaks: Hfq-unbound stable, Hfq-bound stable, and Hfq-bound unstable. (B) Distribution profiles of all CLIP-seq and RNA-seq reads around the center of Hfq-bound strong intergenic peaks. (C) Distribution profiles of all CLIP-seq and RNA-seq reads around the center of Hfq-bound nonstrong intergenic peaks. (D) Dot plot of the abundance of three classes of mRNA peaks: Hfq-unbound stable, Hfq-bound stable, and Hfq-bound unstable. (E) Distribution profiles of all CLIP-seq and RNA-seq reads around the center of Hfq-bound strong mRNA peaks. (F) Distribution profiles of all CLIP-seq and RNA-seq reads around the center of Hfq-bound nonstrong mRNA peaks. (G) Cumulative plot of gene expression abundance. Genes were divided into three groups by Hfq binding: Hfq-bound nonstrong peak genes, Hfq-bound strong peak genes, and Hfq-unbound genes.

To further explore the above hypothesis, we separated sRNA peaks into three different classes. Stable non-Hfq-bound sRNA peaks (type I) have RNA-seq peaks only and have 156 sRNA members. Unstable Hfq-bound sRNA peaks (type II) have Hfq-bound peaks only and have 361 members. Stable Hfq-bound sRNA peaks (type III) have both Hfq-bound and RNA-seq peaks that overlapped by at least one nucleotide and have 93 members (Table S5). Please note that we described sRNA peaks instead of the whole sRNA transcripts here, and we were detecting the peaks from the same sRNA by RNA-seq and CLIP-seq approaches. We found that these three classes of sRNA peaks have very distinct transcript abundance recovered from RNA-seq data (Fig. 5A). We plotted the length distribution of these three classes of peaks, showing the overlapped peaks were generally longer than the nonoverlapped peaks (Fig. S4A, *P* value < 0.001 by *t* test).

10.1128/mSystems.00245-19.4 FIG S4 Characterization of sRNAs bound by Hfq. (A) Violin plot of the length distribution of CLIP-seq and RNA-seq peaks. Peaks were divided into three classes: CLIP-specific, RNA-specific, and Overlap. (B) Read distribution and Northern blot of three type sRNAs classified by Hfq binding. The top left three panels represent Hfq-unbound stable sRNAs (type I), the top right three panels represent Hfq-bound unstable sRNAs (type II), and the bottom two panels represent Hfq-bound stable sRNAs (type III). Please note that Northern blot analysis of sRNA level was performed on total RNAs from the Hfq+ (WT) and Hfq− (Hfq deleted) strains. (C) Enrichment of intergenic (left) and antisense (right) Hfq-bound motifs in three different types of peaks, including strong Hfq-bound peaks, all Hfq-bound peaks, and RNA-seq peaks. Bar height represents the enrichment of motifs (−log_10_ *P* value). Download FIG S4, TIF file, 4.4 MB. Copyright © 2019 Han et al. 2019 Han et al. This content is distributed under the terms of the Creative Commons Attribution 4.0 International license.

10.1128/mSystems.00245-19.9 TABLE S5 Table of sRNAs identified from the Hfq-FLAG strain. The table merged the CLIP-seq and RNA-seq peaks as a union set. Download Table S5, XLSX file, 0.04 MB. Copyright © 2019 Han et al. 2019 Han et al. This content is distributed under the terms of the Creative Commons Attribution 4.0 International license.

We then plotted CLIP-seq and RNA-seq reads around the center of strong Hfq-bound sRNA peaks to study the RNA abundance around Hfq-bound sites. Interestingly, the distribution of RNA-seq reads inside and downstream of Hfq-bound sites strongly declined compared with that of the upstream (Fig. 5B), which supported the hypothesis of Hfq-induced destabilization of sRNA segments downstream of the Hfq-bound sites. We then plotted CLIP-seq and RNA-seq reads around the center of nonstrong intergenic CLIP peaks, a similar declined abundance of RNA-seq reads was observed downstream of the Hfq-bound sites (Fig. 5C). Interestingly, a highly abundant transcript peak upstream of the Hfq-binding center was observed for nonstrong intergenic CLIP peaks, with a distance of about 120 nt (Fig. 5C).

The distribution of Hfq-bound cDNA reads and transcript cDNA reads in individual sRNA peaks were plotted, showing examples of three classes (Fig. S4B). We also analyzed the stability of these sRNAs in response to Hfq deletion. Northern blot analysis of sRNAs in WT and ΔHfq strain (Fig. S4B) showed that the knockout of Hfq decreased the stability of almost all Hfq-bound sRNAs, regardless of their differential stability in the Hfq+ strain. In contrast, the abundance of all non-Hfq-bound sRNAs was not affected by Hfq deletion (Fig. S4B). These results are consistent with a model where the destabilization of Hfq-bound sRNA segments depends on their base pairing with target mRNAs facilitated by Hfq binding (32). The relationship between Hfq binding and sRNA destabilization suggested that the stable non-Hfq-bound sRNA may lack Hfq-binding motifs. Analysis of the overrepresented motifs in all 373 sRNA peaks identified from RNA-seq revealed the lack of typical Hfq-binding motifs in sRNAs (Fig. S4C).

Destabilization of Hfq-bound sRNAs could have resulted from coupled degradation of a sRNA and its mRNA targets (32). The transcript abundance of the three classes of mRNA peaks were similar to those of sRNAs (Fig. 5D). We then analyzed the distribution of transcript reads around the center of Hfq-bound sites from strong and weak CLIP peaks recovered from mRNA regions. Strong Hfq binding correlated with the destabilization of the downstream mRNA segments, highly similar to that of sRNAs (Fig. 5E). The RNA-seq read distribution upstream of weak Hfq binding sites was almost the same as that of sRNAs, and the downstream destabilization was also evident (Fig. 5F).

In light of the proposed mechanism of Hfq-facilitated sRNA-mRNA degradation, we explored the relationship between Hfq binding of mRNA and their stability. The cumulative abundance of mRNAs displaying strong or weak Hfq peaks was plotted, showing that mRNAs showing weak peaks were more abundant than those showing strong peaks (*P* value = 0.01 by the Kolmogorov-Smirnov [K-S] test; Fig. 5G). Genes displaying non-Hfq-bound peaks were generally expressed at lower levels than those showing Hfq-bound peaks (*P* value < 2.2e−16 by K-S test; Fig. 5G).

### Hfq regulates the processing and/or stability of Hfq-bound sRNAs.

**FIG 6:**
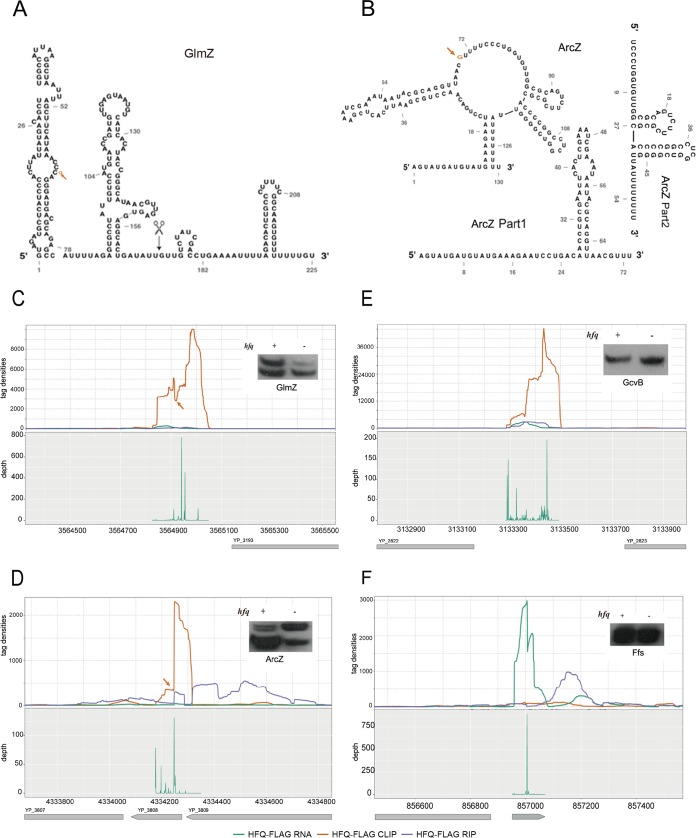
Hfq generally regulates the processing of Hfq-bound sRNAs. (A) Secondary structure of the Y. pestis GlmZ sRNA. The position of the orange arrow represents the processing sites by RNase E. The black scissor represents the reported processing site from E. coli. (B) Secondary structure of the ArcZ sRNA and the two parts after processing. The position of the orange arrow represents the processing sites by RNase E. ArcZ Part1 and ArcZ Part2 represent the secondary structure after processing. (C to F) Distribution of sequencing reads (top panels) and 5′-end site density (bottom panels) of four sRNAs. Northern blot results were also shown in the figure. The orange arrow points to the RNase E cleavage sites, corresponding to the shift site of Hfq-binding peaks. GcvB and Ffs show no processing sites.

Several Hfq-binding sRNAs, including GlmZ and ArcZ, are reported to undergo RNase E processing (36), while GcvB contains two transcriptional termination sites to produce two sRNA isoforms (51–55). The proposed secondary structure of Y. pestis GlmZ is almost identical to that of E. coli (Fig. 6A), while that of ArcZ was strikingly different (Fig. 6B) (55). To provide processing evidence of Hfq-bound sRNAs, we recovered the 5′-end position and density of transcripts from Hfq-FLAG strains using a high-throughput sequencing method (see Materials and Methods for detailed information). The RNase E processing sites on GlmZ and ArcZ were readily recognized from the Hfq binding density map: the CLIP-seq read density cleft for GlmZ (Fig. 6C) and the site indicating sharp read density switch for ArcZ (Fig. 6D). The two sRNA isoforms indicative of cleaved product and uncleaved precursor RNA for both GlmZ and ArcZ were detected by Northern blot analysis (Fig. 6C and D, insets).

Knocking out Hfq led to the change of the ratio between the cleaved products and precursors (Fig. 6C and D), suggesting that Hfq may regulate the processing of these two sRNAs. However, we could not exclude the possibility that Hfq may affect the stability of the cleaved products and precursor differentially. In both GlmZ and ArcZ cases, processing resulted in a U-rich 5′-end (Fig. 6A and B), which was coordinate with the cleavage feature of RNase E (36). Interestingly, the GcvB sRNA did not display two transcript isoforms, although its sequence is highly similar to E. coli and we detected three dominant 5′ sites (Fig. 6E). Ffs also had only one transcript isoform (Fig. 6F). Hfq binding regulation of sRNA stability was also found from other three sRNAs, including sR128, sR142, and sR132 with changed isoform ratios upon Hfq deletion (Fig. S4B).

## DISCUSSION

Sm proteins are a family of small proteins that assemble the core components of the U1, U2, U4, and U5 snRNPs, and therefore are central for eukaryotic pre-mRNA splicing (56). Lsm proteins containing the “Sm motif” often function in eukaryotic mRNA decapping and decay (57, 58). Hfq has been known for more than a half century and is a typical LSm protein (3). By cooperation with diverse sRNAs, Hfq has been shown to play a key role in degrading bacterial mRNAs. The process involves the recruitment of RNase E, a key member of RNA degradosome (7, 30). Decay of mRNA can be either coupled with sRNA or not (4, 5, 59). There are several fundamental questions waiting to be addressed in bacteria, including the following. (i) How many sRNAs and mRNAs are contacted by Hfq? (ii) How do the different Hfq surfaces contact sRNA and mRNA in bacteria? (iii) How does the Hfq binding contribute to sRNA and mRNA base pairing and their decay in bacteria? In this study, we obtained Hfq-bound RNAs by using both CLIP-seq and RIP-seq techniques. By setting RNA-seq data as controls and developing proper algorithm to analyze the genome-wide sequencing data, we were able to address these three questions to a good depth and propose a model for Hfq binding and facilitation of sRNA-mRNA-coupled degradation in bacteria.

### Hfq extensively binds mRNAs.

Hfq is known to play a role in mRNA degradation in E. coli. It interacts with poly(A) polymerase I and is used for substrate recognition by binding to rho-independent terminators (60). It is believed to destabilize that structure and allow polyadenylation to occur. Although several coimmunoprecipitation studies have revealed that Hfq binds hundreds of mRNAs and tens of sRNAs in both E. coli and *Salmonella* (28, 29, 40, 41, 61, 62), more information on Hfq-bound RNAs in other bacteria is needed to better understand Hfq actions. We expressed Hfq-FLAG protein with a Hfq knockout background in Y. pestis and obtained high-quality CLIP-seq and RIP-seq data. By using non-Hfq-bound 23S rRNA as a control, we found that thousands of expressed mRNA genes (∼80%) showed Hfq binding density above background. These results lead to a hypothesis that Hfq might control the stability of most mRNAs with its sRNA partners.

### Hfq flexibly contacts sRNA and mRNAs with multiple surfaces: formation of Hfq-sRNA-mRNA complex.

*In vitro* studies have revealed that Hfq uses its proximal face to bind poly(U) sequence in sRNAs and its distal and rim surfaces to contact A-rich and UA-rich sequences in sRNA and mRNAs (11). Hfq-bound RNA motifs from our CLIP-seq data revealed a comprehensive Hfq binding strategy in cells. In addition to A-rich and UA-rich motifs, Hfq-bound G-rich and UG-rich motifs have been identified in mRNAs of Y. pestis. These motifs mirror A-rich and UA-rich sequences and may be contacted by the distal and rim surfaces of Hfq. For sRNAs, the top three motifs were featured either by the canonical terminator sequence containing the U_6_ stretch motif preceded by the GC-rich sequence (Table 1) or by other two motifs that are G rich or A rich.

The *in vivo* Hfq motifs are comprised of different known short motifs, enabling an Hfq hexamer to use different surfaces to recognize and effectively contact a RNA sequence. The combinatory organization of different motif blocks could allow a specific RNA sequence to contact multiple faces of an Hfq hexamer. This organization could have additional advantages in the assembly of the Hfq-RNA complex. Increasing numbers of sRNAs have been proved to simultaneously act on multiple mRNAs. Likewise, many mRNA transcripts are emerging as shared targets of multiple cognate sRNAs. Since Hfq levels are assumed insufficient relative to RNA species, RNA is shown to actively cycle by competition for the access to Hfq (10, 63).

**FIG 7:**
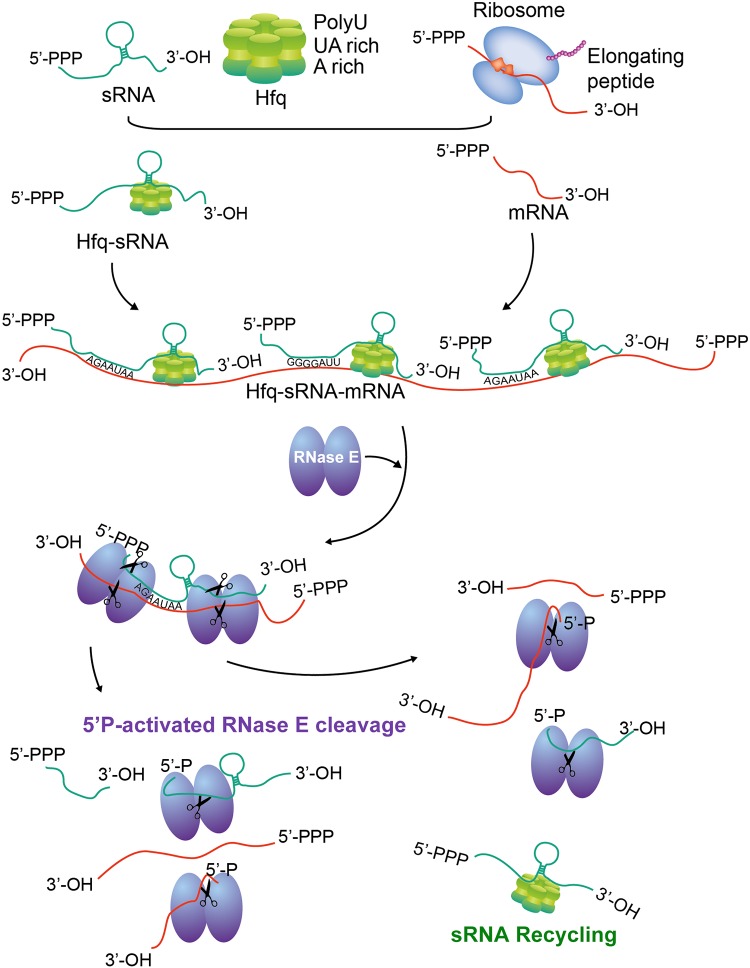
Model presentation of the formation of Hfq-sRNA-mRNA complex, the coupled cleavage of both sRNA and mRNA by RNase E, and the recycling of sRNAs.

The findings presented here expanded our understanding of the dynamics and efficiency of Hfq binding in mRNAs. The results presented in this study suggest that the Hfq-sRNA complexes could select their target in a relative very flexible way. For example, the ubiquitous U_6_ stretch of many sRNAs can base pair with many mRNAs containing A-rich or G-rich motifs at the terminal parts or the body regions. The Hfq-sRNA complex interacts with the translationally inactive and/or repressed mRNAs, which enables the formation of intermolecular base pairing between sRNA-mRNAs and the increase in local concentrations of RNase E for cleavage of sRNA-mRNA duplex (Fig. 7).

### Hfq-bound sRNAs were generally unstable: a comprehensive list of Y. pestis sRNAs.

We have demonstrated that Hfq binding of sRNAs is more complex than expected. First, the predicted sRNAs from RNA-seq data can represent only a population of sRNAs, not all of the sRNAs. Second, a large population of sRNAs are unbalanced in their stability, with the 5′ portion being more stable than the 3′ portion, largely due to the Hfq binding. Therefore, some sRNAs predicted from transcriptome reads may be shorter than the full transcripts. At last, Hfq binding is associated with sRNA degradation during the normal growth condition where transcription is active.

In summary, sRNAs are highly dynamic in their transcription and degradation. Identification of full-length sRNA genes is challenging. The challenge is further complicated by the lack of canonical terminators in many sRNAs and the presence of sRNAs overlapping the 3′ ends of mRNA genes (42, 44, 64). In this study, we generated a comprehensive sRNA list comprising about 700 members encoded by avirulent Y. pestis strains; only a small fraction of these have been identified before. This list does not include those that overlapped the 3′ region of mRNA genes. About 363 Hfq-bound sRNAs difficult to be identified by transcriptome sequencing were identified. We therefore proposed the idea that Hfq binds hundreds of sRNAs, which could be involved in controlling the stability and translation of most, if not all, mRNAs in Y. pestis.

### Hfq-bound sites define two positions for the coupled degradation of the base-paired sRNA-mRNA: a general mechanism for cellular mRNA surveillance.

The mechanism of the coupled degradation of the sRNA-mRNA complex appears feasible for a quick response of environmental change by bacterial cells (7) and was proposed in 2003 (32), but there is little direct evidence supporting it (4, 7, 30). In this study, genome-wide analysis of multiple classes of sRNAs and mRNAs in their aspects of Hfq-bound capability allows us to comprehensively revisit this issue. We have demonstrated that sRNA segments at Hfq-bound sites and downstream of these sites are globally unstable. The mRNAs displaying strong Hfq-bound peaks showed a pattern similar to the pattern that Hfq-bound sRNA showed. However, mRNAs displaying nonstrong Hfq-bound peaks showed that the Hfq-binding sites are protected, while the downstream segments are destabilized. In light of the RNase E function in Hfq-sRNA-mediated RNA degradation (30), we proposed that Hfq-bound sites render two positions for RNase E entry, which will result in the mRNA segment degradation downstream of Hfq binding sites via a 5′P-dependent RNase E degradation pathway (Fig. 7). This hypothesis is in line the current knowledge of Hfq in regulating RNA stability via interaction with poly(A) polymerase I (65). The coupled degradation of both mRNA and sRNA in the Hfq-bound sites lead to either the direct degradation of the 5′-P-containing sRNAs or recycling of cleaved sRNAs containing either 5′-P or 5′-PPP by RNase E cleavage. Recycling of cleaved sRNAs explains the lack of protected Hfq-bound sites in sRNAs. The U-rich binding sites of Hfq also suggest that degradation can also occur when Hfq binds to rho-independent terminators to trigger polyadenylation (60).

Interestingly, we showed that Hfq-bound AGAAUAA motifs are located at both the 5′ and 3′ termini of Y. pestis and E. coli mRNAs, while CUUGGG and GGGAUU are located at the body regions of mRNAs. All these sequence motifs are partially complementary to U-rich sequence in sRNAs. Given the large diversity of sRNA sequences, it is not surprising that the Hfq-sRNA complex has a chance to bind most of cellular mRNAs and to mediate their degradation when they are not effectively translated. Although there are Hfq-bound mRNA motifs, we find that Hfq binding of mRNAs lacks selectivity because the motifs were intrinsic features of mRNAs. The lack of binding site selection supports the RNA chaperone Hfq surveilling the RNA homeostasis of the whole bacterial transcripts via the cooperation with its partner sRNAs.

## MATERIALS AND METHODS

### Construction of Flag-tagged plasmids.

By using a fusion PCR protocol, oligonucleotides encoding 3×FLAG affinity tag (DYKDHDGDYKDHDIDYKDDDDK) were added before the TAA termination codon of the Hfq gene to construct the C-terminally Flag-tagged plasmids. A fragment covering a region of 298 nucleotides (nt) upstream, the entire *hfq* gene followed by *3*×*flag* and 176 nt downstream was cloned into the multiple cloning site of plasmid pACYC184, designated pHfq-FLAG. The other fragment spanning a region of 298 nt upstream, the first 21 nt of the *hfq* gene followed by *3*×*flag* and 176 nt downstream was also introduced into pACYC184, designated FLAG. The complementary plasmid was constructed by inserting a PCR fragment covering a region from the 300-bp fragment upstream to 200 bp downstream of the *hfq* gene into pACYC184, designated pHfq. The inserts mentioned above were cloned into pACYC184 via BamHI and XbaI/EcoRV restriction sites. The list of oligonucleotide primers was shown in Table S6 in the supplemental material.

10.1128/mSystems.00245-19.10 TABLE S6 Oligonucleotide primers used for plasmid construction. Download Table S6, DOCX file, 0.01 MB. Copyright © 2019 Han et al. 2019 Han et al. This content is distributed under the terms of the Creative Commons Attribution 4.0 International license.

### Bacterial strains and growth conditions.

Y. pestis wild-type strain 201 belongs to a newly established Y. pestis biovar, microtus, which is avirulent in humans but highly lethal in mice. The *hfq* deletion strain (Δ*hfq*) was generated by λ-Red homologous recombination methods as previously described (38). The Y. pestis Hfq-FLAG, Hfq, and WT-FLAG strains were constructed by transforming the pHfq-FLAG and pHfq into the Δ*hfq* strain and Flag into the WT strain, respectively. Bacteria were grown in brain heart infusion (BHI) broth (Difco) supplemented with appropriate antibiotics overnight at 26°C with shaking at 200 rpm until exponential growth phase (optical density at 629 nm [OD_620_] of 0.8). Bacterial growth was stopped by centrifugation for 6 min at 5,000 rpm at 4°C. The pellets were frozen into liquid nitrogen and stored at −80°C until the cells were lysed. Western blotting was performed by using monoclonal FLAG antibody (Sigma) to detect the FLAG-tagged proteins.

### RNA-seq, CLIP-seq, and RIP-seq.

For RNA-seq, total RNAs were extracted from Y. pestis Hfq-FLAG, WT-FLAG, and Hfq strains mentioned above by using TRIzol reagent (Invitrogen). For CLIP-seq, two strains with FLAG grown under the same conditions were collected and resuspended in 10 mM Tris-HCl (pH 8.0). The pellets were dispersed on a petri dish and irradiated uncovered with 400 mJ/cm^2^ of UV 254 nm to form the cross-linked RNA-protein complex. Bacterial cells were collected and lysed in RIP lysis buffer (1× phosphate-buffered saline [PBS], 0.1% SDS, 0.5% NP-40, and 0.5% sodium deoxycholate) and subjected to coimmunoprecipitation (Co-IP). Co-IP was conducted to isolate the FLAG-bound and Hfq-FLAG-bound RNA by using FLAG antibody according to the manufacturer’s instructions for RNA-binding protein immunoprecipitation kit (Millipore). Briefly, the lysate was centrifuged at 12,000 × *g* at 4°C for 10 min. The clear lysate was incubated with 1.0 ml of bead-antibody complex in RIP immunoprecipitation buffer, followed by incubation at 4°C for 3 h on a rotator. The immunoprecipitation tubes were centrifuged briefly and placed on the magnetic separator, and the supernatant was discarded. The anti-FLAG beads were then washed a total of six times with 0.5 ml of RIP wash buffer and digested by RNase T. The immunoprecipitated RNA fragments were radiolabeled using PNK and separated by SDS-PAGE. The bands corresponding to the equivalent size of Hfq protein were cut out and purified. The cross-linked RNA-protein complexes were digested with proteinase K at 55°C for 30 min. RNA was extracted using TRIzol and phenol-chloroform, followed by isopropanol precipitation. The purified RNA was treated with DNase I (Promega) and sequenced using the Illumina/Solexa RNA-sequencing protocol.

For RIP-seq, 500 μl lysate was incubated with 10 μg anti-Flag antibody or control IgG antibody overnight at 4°C. The immunoprecipitates were further incubated with protein A Dynabeads for 1 h at 4°C. After applying to magnet and removing the supernatants, the beads were sequentially washed with lysis buffer, high-salt buffer (250 mM Tris [pH 7.4], 750 mM NaCl, 10 mM EDTA, 0.1% SDS, 0.5% NP-40, and 0.5 deoxycholate), and PNK buffer (50 mM Tris, 20 mM EGTA, and 0.5% NP-40) two times in each buffer. The immunoprecipitates were eluted from the beads with elution buffer (50 nM Tris [pH 8.0], 10 mM EDTA, and 1% SDS), and the RNA was purified with TRIzol reagent (Life Technologies).

Purified RNAs were iron fragmented at 95°C followed by end repair and 5′ adaptor ligation. Then reverse transcription was performed with reverse transcriptase (RT) primer harboring 3′ adaptor sequence and randomized hexamer. The cDNAs were purified and amplified, and PCR products corresponding to 200 to 500 bp were purified, quantified and stored at −80°C until used for sequencing.

For high-throughput sequencing, the libraries were prepared following the manufacturer’s instructions and applied to Illumina GAIIx system for 80 single-end sequencing by ABLife Inc., Wuhan, China.

### RNA 5′-end library construction.

Total RNA was treated with RQ1 DNase (Promega) to remove DNA. The quality and quantity of the purified RNA were determined by measuring the absorbance at 260 nm (*A*_260_)/*A*_280_ using SmartSpec Plus (Bio-Rad). RNA integrity was further verified by 1.5% agarose gel electrophoresis. A total of 2.5 μg of total RNA was used for cDNA library preparation with the adaptors in accordance with Illumina protocol. Briefly, RNAs were ligated to 3′ and 5′ adaptors sequentially and reverse transcribed to cDNA and then PCR amplified (RNA 3′ adaptor, 5rApp/ATCTCGTATGCCGTCTTCTGCTTG/3′ NH_2_/, RNA 5′ adaptor, 5′GUUCAGAGUUCUACAGUCCGACGAUCNNN3′). The whole library was applied to 10% native polyacrylamide gel electrophoresis, and bands corresponding to 80- to 380-bp RNA insertion were cut and eluted. After ethanol precipitation and washing, the purified RNA libraries were quantified with Qubit fluorometer (Invitrogen) and used for cluster generation and 36-nt single-end sequencing analysis by using the Illumina GAIIx (Illumina, San Diego, CA, USA) according to the manufacturer’s instructions.

### Raw sequencing data filtering and gene expression calculation.

Raw reads were first discarded if containing more than 2-N bases. The reads were then processed by clipping adaptor sequences and removing low-quality bases (less than 20) by using FASTX-Toolkit (version 0.0.13). After that, filtered reads (>13 nt) were aligned to the Y. pestis 91001 reference genome (66) by bowtie2 (67) with no more than 1 seed mismatch. Aligned reads with more than one genome location were discarded due to their ambiguous locus. Uniquely localized reads were used to do the following analysis. For RNA-seq data, the RPKM (reads per kilobase of transcript per million mapped reads) value of each gene was obtained from the uniquely aligned reads.

### Peak calling method and sRNA definition.

To have an exact prediction of Y. pestis sRNA and Hfq binding site, we developed a window-based algorithm to detect peaks from alignment results among intragenic, intergenic, and antisense regions. A 5-bp window size was chosen as the default window size. The peak starting site was defined as the end of one window whose median depth is lower than one quarter depth from any one of the eight downstream adjacent windows. The peak terminal site was defined as the start of one window whose median depth is lower than one quarter depth from any one of the eight upstream adjacent windows. After obtaining the original peaks, we then filtered the peaks according to the following three thresholds: (i) the length of peaks should range from 40 bp to 500 bp; (ii) the maximum height of one peak should be no less than 60; (iii) the medium height of one peak should be no less than 20 nt. After peak definition, we classified the peaks into three different classes according to their locations: (i) intragenic peaks were defined as peaks whose locus overlapped with known mRNA genes on the same strand; (ii) antisense peaks were defined as peaks whose locus overlapped with known mRNA genes on the opposite strand; (iii) intergenic peaks were defined as neither intragenic nor antisense peaks. Antisense and intergenic peaks were defined as sRNAs both in RNA-seq and CLIP-seq samples.

We performed the peak calling method for both CLIP-seq and RNA-seq. According to the peak location, we classified peaks into three types. Stable Hfq-unbound peaks (type I) refer to those having RNA-seq transcript peaks only. Unstable Hfq-bound peaks (type II) refer to those having Hfq-bound peaks only. Stable Hfq-bound peaks (type III) refer to those with both Hfq-bound and transcript peaks that overlapped at least one nucleotide (93 members) (Table S5).

### Hfq-bound strong peak definition.

The strategy of identifying strongly Hfq-bound and -unbound mRNAs and sRNAs was described as follows. Briefly, peaks identified from the above strategy were the candidate bound and unbound site from Hfq-FLAG CLIP and Hfq-FLAG RNA samples. The total number of mapped reads within each peak was calculated. The sequencing data were displayed with the relative depth of mapped reads at each position of nucleotides on the global-genome scale. A fourfold threshold of total base in each peak between CLIP-seq and RNA-seq was used to define the Hfq-bound or -unbound peaks. The corresponding peak genes were defined as the Hfq-bound or non-Hfq-bound genes.

### Motif search and distribution.

To illustrate the binding nucleotide pattern of Hfq, we searched the RNA motif enrichment by Homer software (46). Then we realigned the top motifs to the peak sequence by fuzznuc (http://emboss.sourceforge.net/apps/release/6.2/emboss/apps/fuzznuc.html), and motif distribution was plotted by the normalized lengths of peaks.

### Northern blot.

A DIG Northern Starter kit (Roche) was used to perform Northern blotting according to the manufacturer’s protocol as previously described (68). Pure bacterial cultures were mixed with RNAprotect bacterial reagent (Qiagen) to minimize RNA degradation. Total RNA was then extracted from bacterial strains using the TRIzol reagent (Invitrogen). Total RNA samples (3 μg) were denatured at 70°C for 5 min, separated on 6% polyacrylamide−7 M urea gel, and transferred onto Hybond N+ membranes (GE) by electroblotting. The membranes were UV cross-linked and prehybridized for 1 h. RNA probes labeled with DIG-11-UTP by *in vitro* transcription using T7 RNA polymerase were added. The membranes were then hybridized overnight at 68°C in a DIG Easy Hyb according to the manufacturer’s protocols. RNA was immunologically detected and exposed to X-ray film. Multiple exposures to X-ray film were taken to achieve the desired signal strength.

### RIP-PCR.

Based on the immunoprecipitation, purified RNAs were treated at 65°C for 5 min. Then reverse transcription was performed with RT primer harboring 3′ adaptor sequence and randomized hexamer. The cDNAs were amplified by 2× Dream *Taq* Mix (Thermo Fisher Scientific), and PCR products were electrophoretically analyzed on agarose gels.

### Other statistical analyses.

In-house script (sogen) was used for visualization of next-generation sequence data and genomic annotations. Circos software (69) was used to illustrate the global Hfq-binding profile. To assess the functional enrichment of a given gene set, we aligned the protein sequence of Y. pestis to the KEGG database. Then we used hypergeometric test to calculate the enrichment of a given gene set, and all genes were regarded as background. R software was used to perform statistical significance analysis, including all the hypothesis testing types in the article.

### Data availability.

The RNA-seq, CLIP-seq, and RIP-seq data reported in this paper have been deposited in NCBI GEO under accession number GSE77555.
