# Supplementary figures and images for: Hfq Globally Binds and Destabilizes sRNAs and mRNAs in Yersinia pestis
Source: mSystems. 2019 Jul 16;4(4):e00245-19. doi: 10.1128/mSystems.00245-19 (PMC6635623; doi:10.1128/mSystems.00245-19)

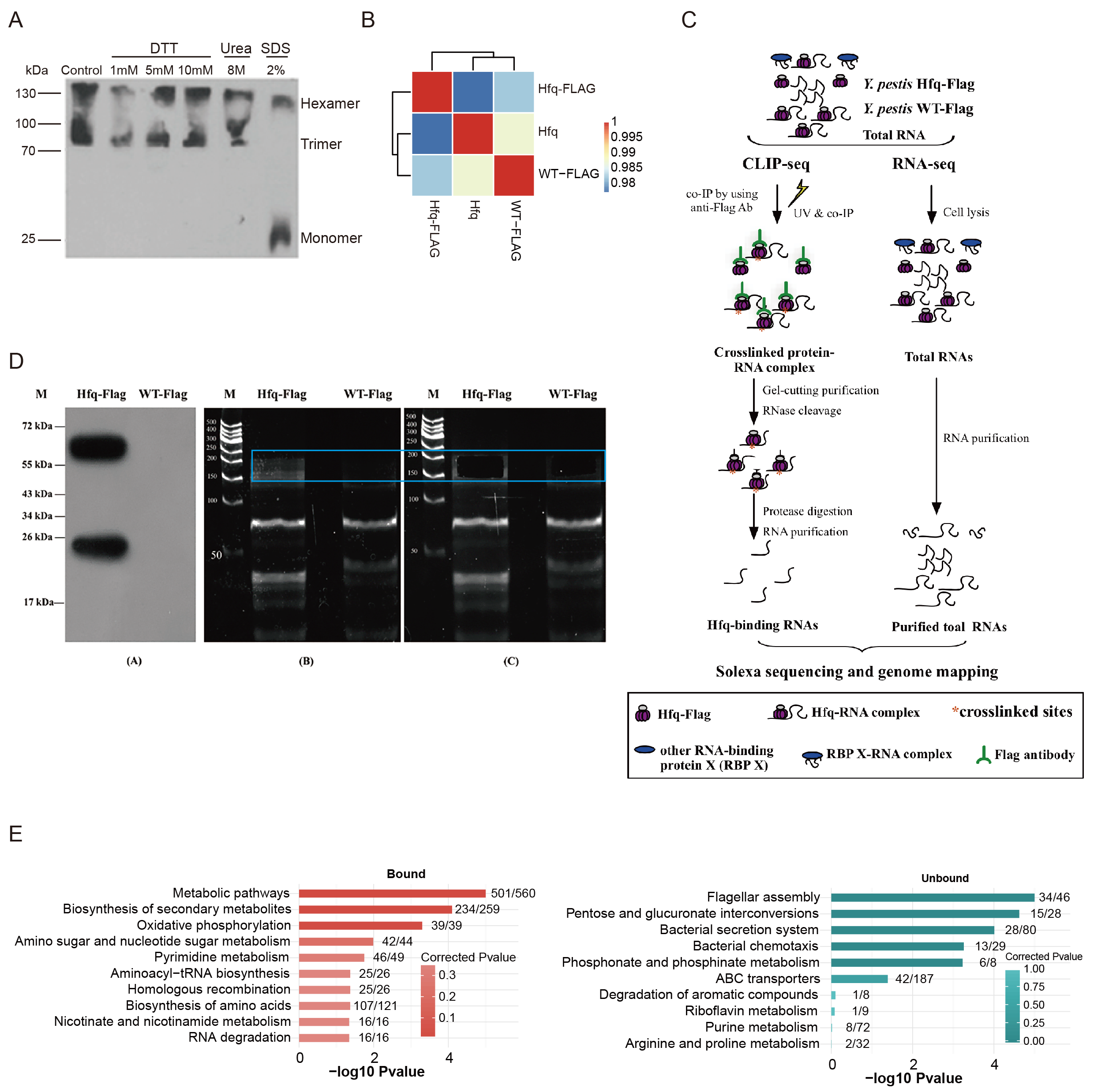

Supplement: FIG S1 [file mSystems.00245-19-sf001.tif]

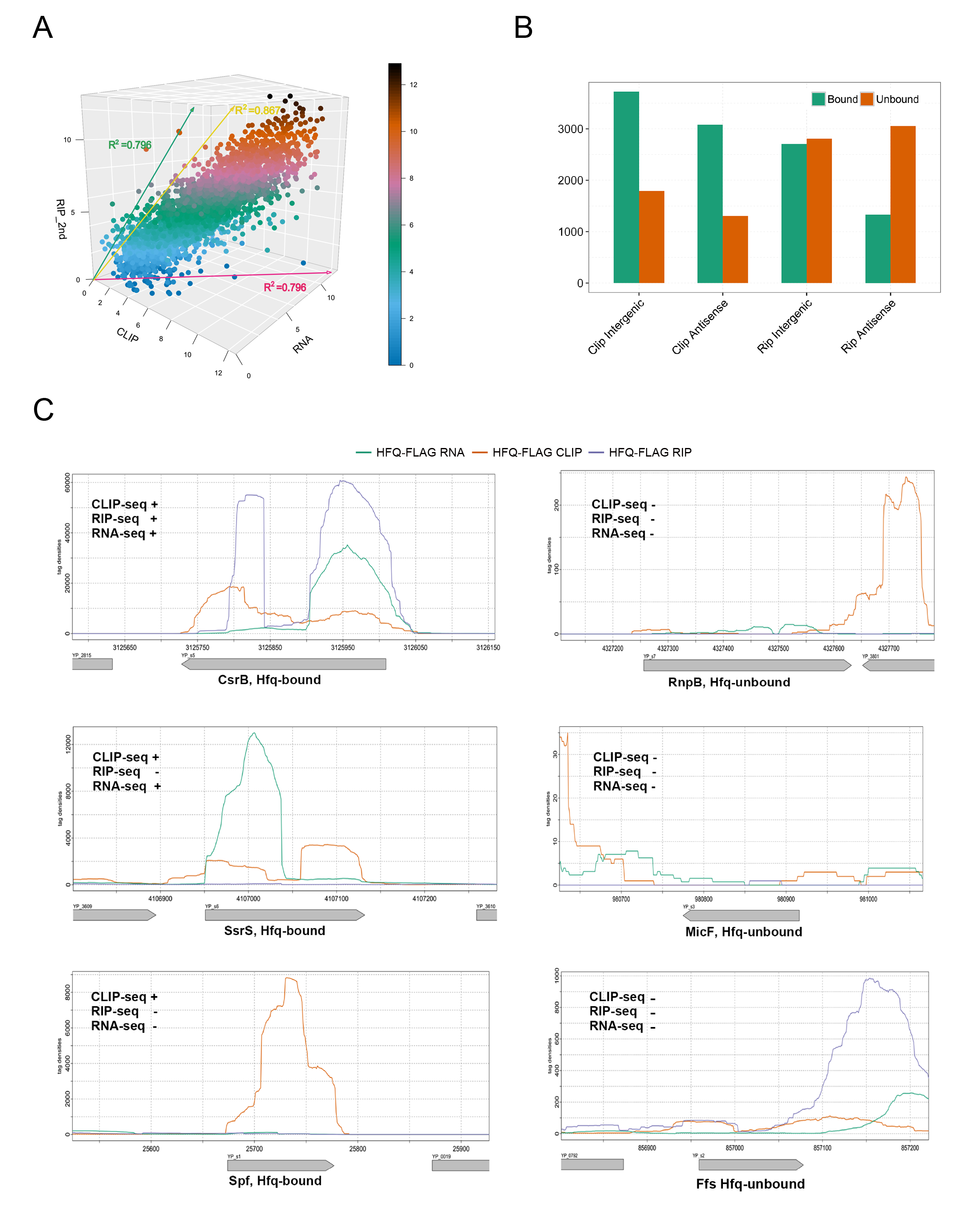

Supplement: FIG S2 [file mSystems.00245-19-sf002.tif]

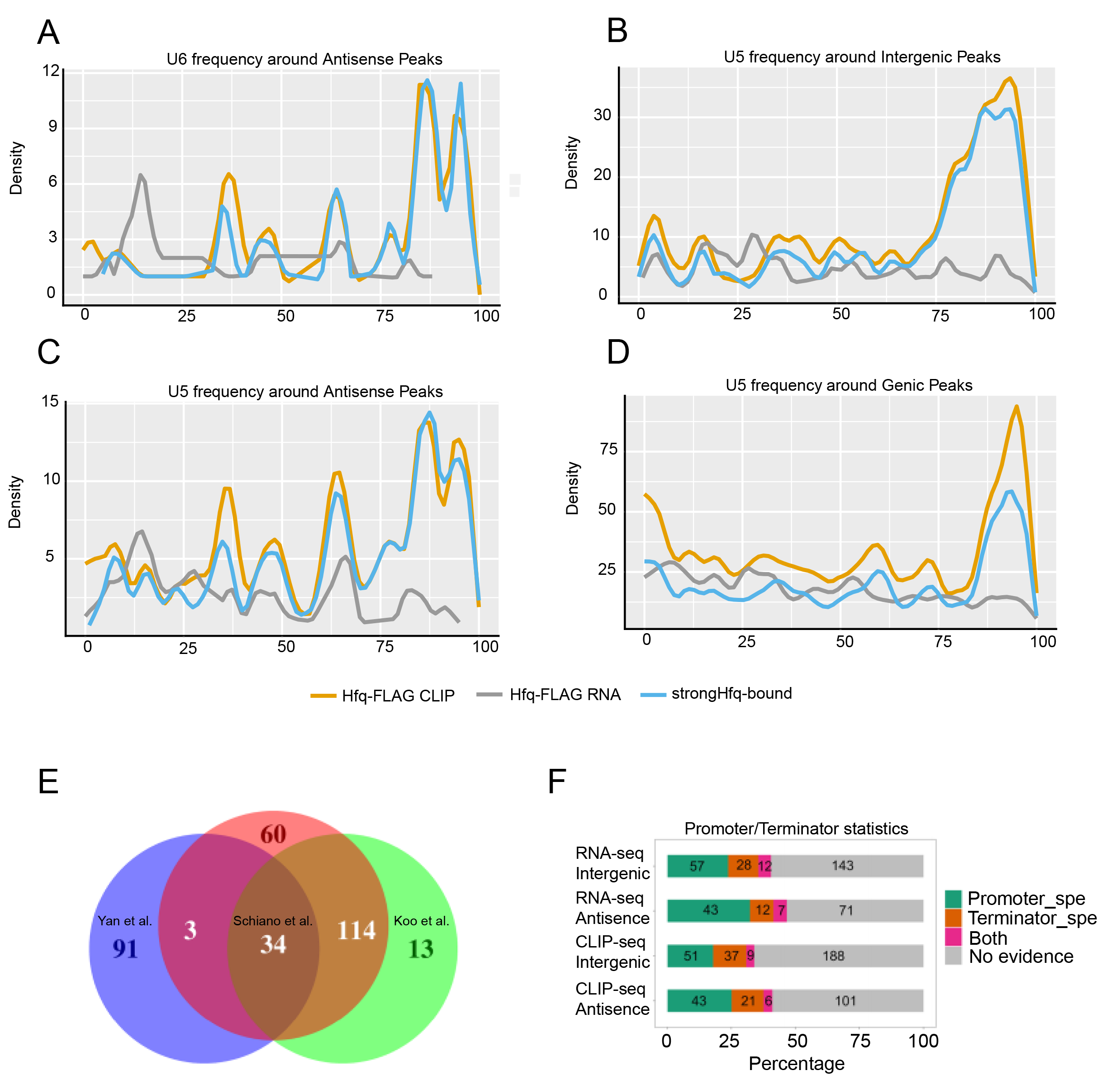

Supplement: FIG S3 [file mSystems.00245-19-sf003.tif]

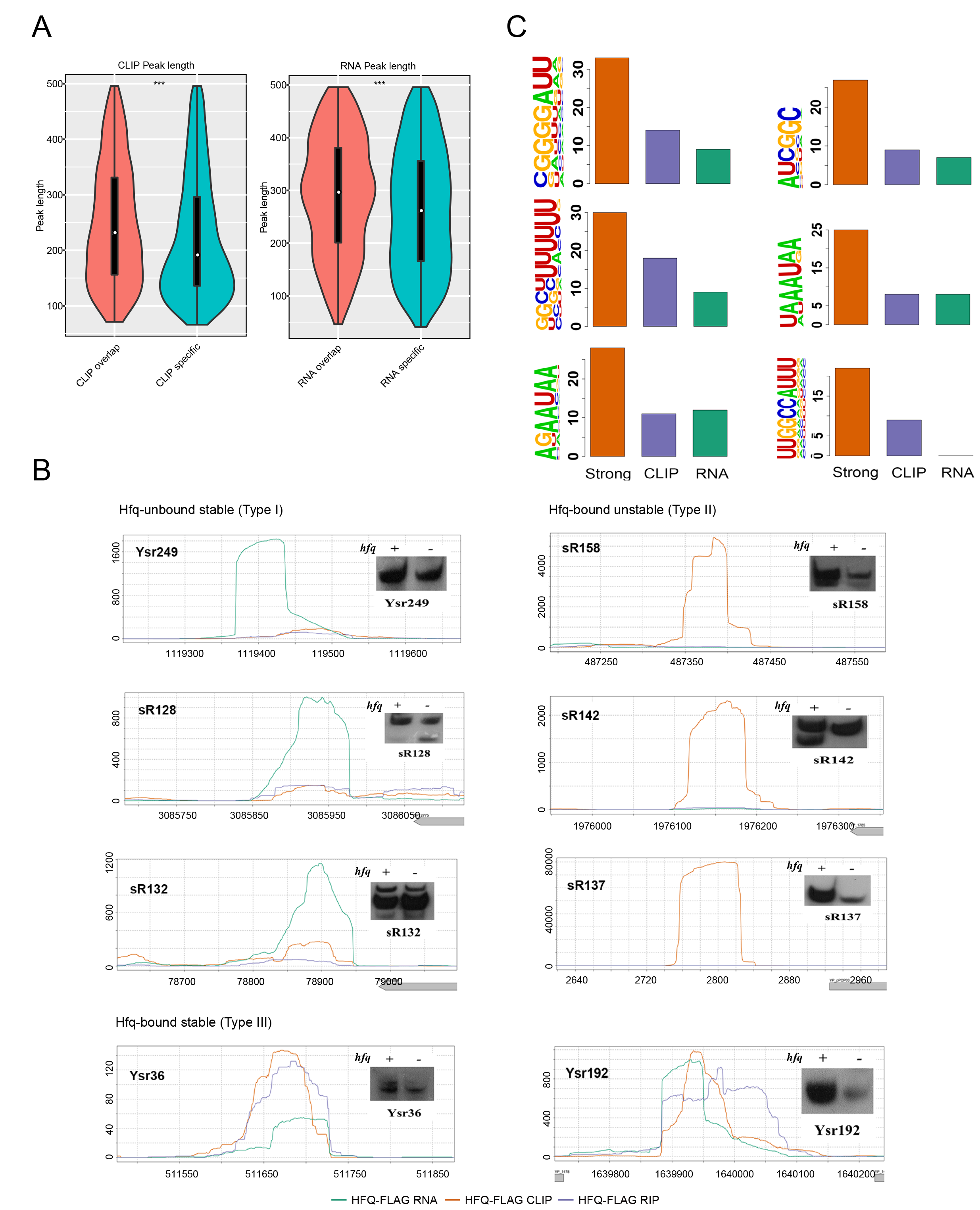

Supplement: FIG S4 [file mSystems.00245-19-sf004.tif]
