# Supplementary material for: Hfq Globally Binds and Destabilizes sRNAs and mRNAs in Yersinia pestis
Source: mSystems. 2019 Jul 16;4(4):e00245-19. doi: 10.1128/mSystems.00245-19 (PMC6635623; doi:10.1128/mSystems.00245-19)
Supplement: TABLE S3 [file mSystems.00245-19-st003.docx]

| **Table S3-Peak summary and classification by region.** | | | | | |
| --- | --- | --- | --- | --- | --- |
| **Sample** | **Total** | **InGene** | **Intergenic** | **Antisense** | **Asso gene*** |
| Hfq-FLAG CLIP | 2511 | 2055 | 285 | 171 | 1619 |
| WT-FLAG CLIP | 46 | 27 | 16 | 3 | 27 |
| Hfq-FLAG RNA | 1518 | 1268 | 164 | 86 | 1095 |
| WT-FLAG RNA | 1731 | 1416 | 206 | 109 | 1154 |
| Hfq RNA | 1447 | 1209 | 159 | 79 | 987 |

* Asso.gene represents the gene number of corresponding peaks.
