# Supplementary material for: Hfq Globally Binds and Destabilizes sRNAs and mRNAs in Yersinia pestis
Source: mSystems. 2019 Jul 16;4(4):e00245-19. doi: 10.1128/mSystems.00245-19 (PMC6635623; doi:10.1128/mSystems.00245-19)
Supplement: TABLE S6 [file mSystems.00245-19-st006.docx]

**Table S6. Oligonucleiotide primers used for plasmid construction**

| **Primer name** | **sequence (5’-3’)** |
| --- | --- |
| Hfq/Flag-F | ATCGGATCCATGTGGTCTTATCTGTCTGGTG |
| Hfq-3×Flag-R | GATATCATGATCTTTATAATCACCGTCATGGTCTTTGTAGTCTTCAGCGTCATCACTGTCC |
| Hfq-3×Flag-F | GACGGTGATTATAAAGATCATGATATCGACTACAAAGATGACGACGATAAATAAAGCCCATTGCTGGTCGAC |
| Hfq/Flag-R | GCTTCTAGAATCCTCTGTGTCTTTGTTTTGC |
| Flag-F | ATGGCTAAGGGGCAATCTTTGGACTACAAAGACCATGACG |
| Flag-R | CAAAGATTGCCCCTTAGCCATTCTATA |
| Hfq-F | ATCGGATCCTTGCCTGCCATTCGTTGTG |
| Hfq-R | GCTGATATCACCAATCCCGCCCTTCTG |
